# Toll-like receptor 9-positive plasmacytoid dendritic cells promote Th17 immune responses in oral lichen planus stimulated by epithelium-derived cathepsin K

**DOI:** 10.1038/s41598-023-46090-3

**Published:** 2023-11-07

**Authors:** Yuka Miyahara, Hu Chen, Masafumi Moriyama, Keita Mochizuki, Naoki Kaneko, A. S. M. Rafiul Haque, Akira Chinju, Kazuki Kai, Mizuki Sakamoto, Noriko Kakizoe-Ishiguro, Masaki Yamauchi, Kenichi Ogata, Tamotsu Kiyoshima, Shintaro Kawano, Seiji Nakamura

**Affiliations:** 1 Section of Oral and Maxillofacial Oncology, Division of Maxillofacial Diagnostic and Surgical Sciences, Faculty of Dental Science, Kyushu University, 3-1-1 Maidashi, Higashi-ku, Fukuoka, 812-8582 Japan; 2 OBT Research Center, Faculty of Dental Science, Kyushu University, Fukuoka, Japan; 3 Department of Dental Anatomy, Udayan Dental College, Rajpara, Bangladesh; 4 Laboratory of Oral Pathology, Division of Maxillofacial Diagnostic and Surgical Sciences, Faculty of Dental Science, Kyushu University, Fukuoka, Japan

**Keywords:** Inflammation, Innate immunity, Lymphocytes, Mucosal immunology

## Abstract

Oral lichen planus (OLP) is a chronic inflammatory disease associated with T cell infiltration. The crosstalk between oral epithelium and mucosal T cells was considered to be crucial in the pathogenesis of OLP. Here, we selectively extracted the normal epithelium (NE) and lesional epithelium (LE) of buccal mucosa specimens from three patients with OLP by laser capture microdissection due to identify the pathogenic factors. Cathepsin K (CTSK) was identified as one of common upregulated genes in the LE by DNA microarray. Immunohistochemically, CTSK was distinctly detected in and around the LE, while it was rarely seen in the NE. Recent studies showed that CTSK enhanced Toll-like receptor 9 (TLR9) signaling in antigen-presenting cells, leading to Th17 cell differentiation. TLR9 expression mainly co-localized with CD123^+^ plasmacytoid dendritic cells (pDCs). The number of RORγt-positive cells correlated with that of CTSK-positive cells in OLP tissues. CD123^+^ pDCs induced the production of Th17-related cytokines (IL-6, IL-23, and TGF-β) upon stimulation with TLR9 agonist CpG DNA. Moreover, single cell RNA-sequencing analysis revealed that TLR9-positive pDCs enhanced in genes associated with Th17 cell differentiation in comparison with TLR9-negative pDCs. CTSK could induce Th17-related production of CD123^+^ pDCs via TLR9 signaling to promote the pathogenesis of OLP.

## Introduction

Oral lichen planus (OLP) is a chronic inflammatory disease characterized by parakeratosis and band-like lymphocytic infiltration in the subepithelial layer of oral mucosa. The most common affected site in OLP is the buccal mucosa (BM) with symmetrical involvement, followed by the lip, gingiva, tongue, and palate^1^. Five clinical forms of OLP are seen: atrophic, bullous, erosive, plaque-like, and reticular. Especially, erosive forms, female gender, and tongue site of OLP was considered as risk factors for malignant transformation^2,3^. Therefore, the World Health Organization working group has categorized OLP as an oral potentially malignant disorder^4,5^.

Regarding the immunological aspects of OLP, the dominant population in the subepithelial-infiltrating lymphocytes is CD4^+^ T helper (Th) cells, especially Th2 and Th17 cells^6–8^. Th2 cytokine levels are significantly increased in both peripheral blood and saliva from OLP patients^9,10^. Another study showed that the number of *Prevotella* bacteria was increased in the oral cavity of OLP patients and is associated with enhanced Th17-mediated mucosal inflammation^11^. Furthermore, the serum concentration of IL17A in patients with OLP was increased compared with that in sex-age-matched healthy controls^12^. Several studies have demonstrated that the crosstalk between these specific Th subsets and lesional epithelium (LE) was involved in the pathogenesis of OLP^13,14^. However, the mechanism underlying Th-related inflammation in the LE of OLP remains unknown.

In this study, we selectively extracted the epithelial layer in OLP specimens using laser capture microdissection (LCM) and analyzed samples by DNA microarray analysis to identify disease-associated genes, especially Th-immune molecules.

## Results

### Identification of gene expression patterns in the LE and normal epithelium (NE) from OLP patients.

**Figure 1:**
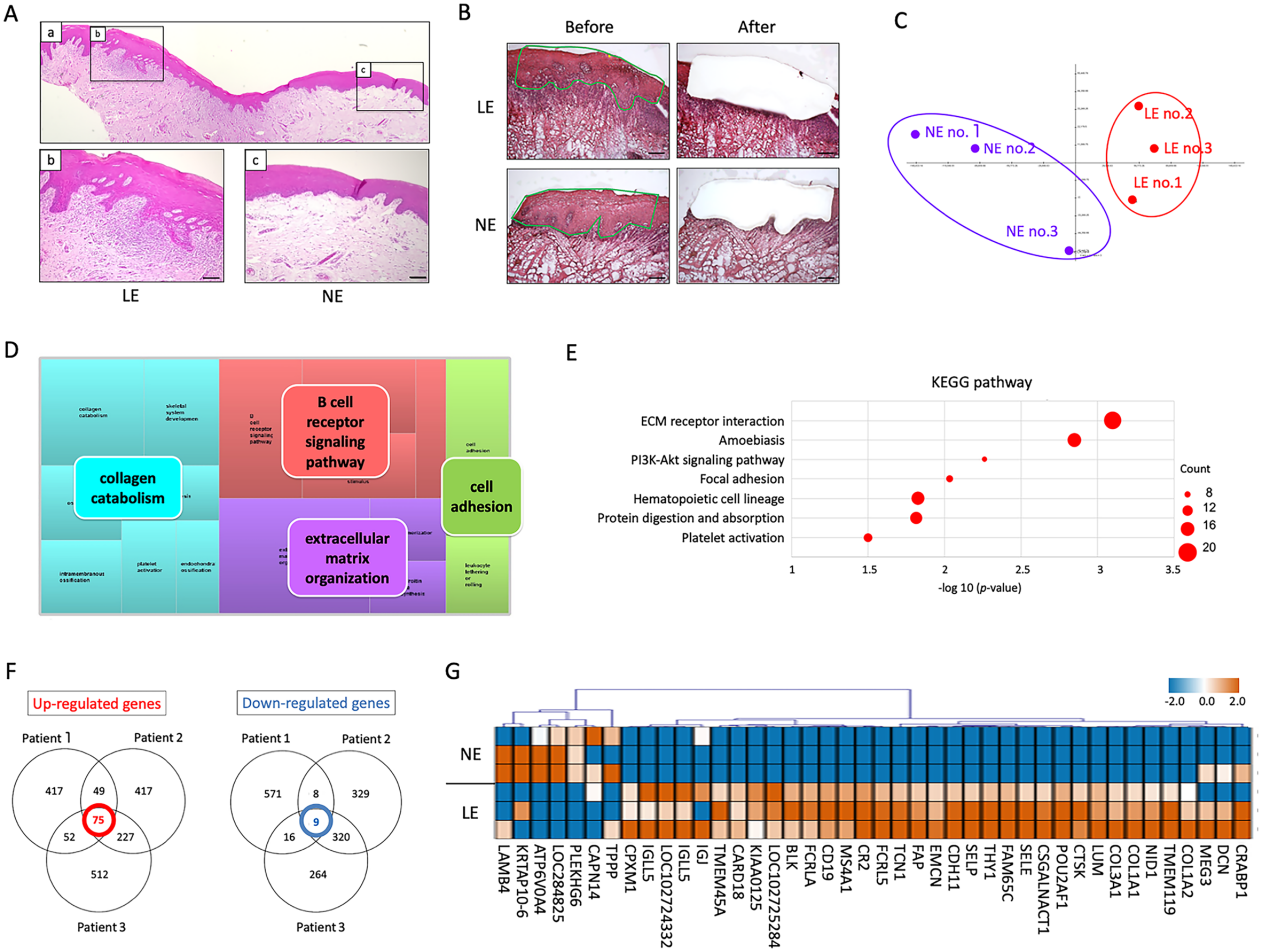
Gene expression patterns in lesional epithelium (LE) and normal epithelium (NE) of buccal mucosa (BM) specimens from patients with oral lichen planus (OLP). (**A**) Representative images of BM specimens from an OLP patient stained with hematoxylin and eosin (a–c). Scale bars, 100 μm. (**B**) Representative images of LE and NE extracted from BM specimens before and after laser capture microdissection. The green line indicates infiltration of inflammatory cells. Scale bars, 100 μm. (**C**) Principal components analysis between the LE and NE from three patients with OLP using the quantile-normalized data. (**D**) TreeMap was generated using enriched Gene Ontology (GO) terms of the biological process of differentially expressed genes (DEGs) in LE compared with NE. The size of the 15 rectangles reflected the significance of the enriched GO term; the terms were divided into four categories (indicated by different colors). (**E**) Enriched Kyoto Encyclopedia of Genes and Genomes (KEGG) pathways of differentially expressed genes (DEGs) upregulated in LE compared with NE. (**F**) Common DEGs in LE compared with NE from three patients with OLP. (**G**) Heat map shows statistically significantly differences in gene expression levels between LE and NE from three patients with OLP.

Figure 1A shows representative images of paraffin sections stained with hematoxylin and eosin (HE). We first selectively extracted the LE and NE from BM specimens of three patients with OLP using LCM; representative findings are shown in Fig. 1B. To determine the gene expression profiles of the extracted LE and NE, microarray analysis of 60,000 genes was performed. Scatterplot analysis was conducted to elucidate and represent values for two different numeric variables between the LE and NE, and the results showed statistically significant differences in gene expression (Supplementary Fig. 2). The gene expression patterns in LE and NE were markedly different by principal components analysis (PCA) (Fig. 1C); the gene expression patterns between the LE and NE were divided into two different clusters. The first principal component (PC1) accounted for 78.7% of data, while PC2 accounted for 12.7%.

The LE was found to have a total of 1371 DEGs compared with the NE from patients with OLP; significantly enriched Gene Ontology (GO) terms were analyzed using DAVID. In TreeMap (a space-filling visualization technique for hierarchical structures), the following 4 categories composed of 15 rectangles were identified as involved in OLP: collagen catabolism, skeletal system development, ossification, skin morphogenesis, intramembranous ossification, platelet activator, endochondral ossification, B cell receptor signaling pathway, leukocyte migration, cellular response to amino acid stimulus, extracellular matrix organization, protein heterodimerization, chondroitin sulfate biosynthesis, immune responses, cell activation, and leukocyte tethering or rolling (Fig. 1D). Kyoto Encyclopedia of Genes and Genomes (KEGG) pathway analysis indicated that differentially expressed genes (DEGs) upregulated in the LE from patients with OLP encoded proteins that function in ECM receptor interaction, amoebiasis, PI3K-Akt signaling pathway, focal adhesion, hematopoietic cell lineage, protein digestion and absorption, and platelet activation (Fig. 1E).

After determining gene expression patterns in the LE and NE from three patients with OLP by clustering analysis, 75 genes were identified as commonly upregulated DEGs in LE by the rank products method, and 9 genes were identified as commonly downregulated DEGs in LE (Fig. 1F). From these 84 common DEGs, 42 DEGs (35 upregulated and 7 downregulated genes) were extracted as protein-coding genes. The 42 DEGs in the three patients with OLP are displayed on the hierarchical clustering heatmaps in Fig. 1G.

The cathepsin K (CTSK) gene, an immune-regulated gene, was identified as one of the common upregulated genes in the LE. CTSK is an osteoclast-specific lysosomal protease^15^. Notably, several studies reported that CTSK promoted Toll-like receptor 9 (TLR9)-induced cytokine production (IL-6, IL-23, and TGF-β) in dendritic cells (DCs), which play important roles in the induction and expansion of Th17 cells^16,17^. Therefore, we focused on CTSK in subsequent experiments.

### Expression of CTSK in OLP tissues

**Figure 2:**
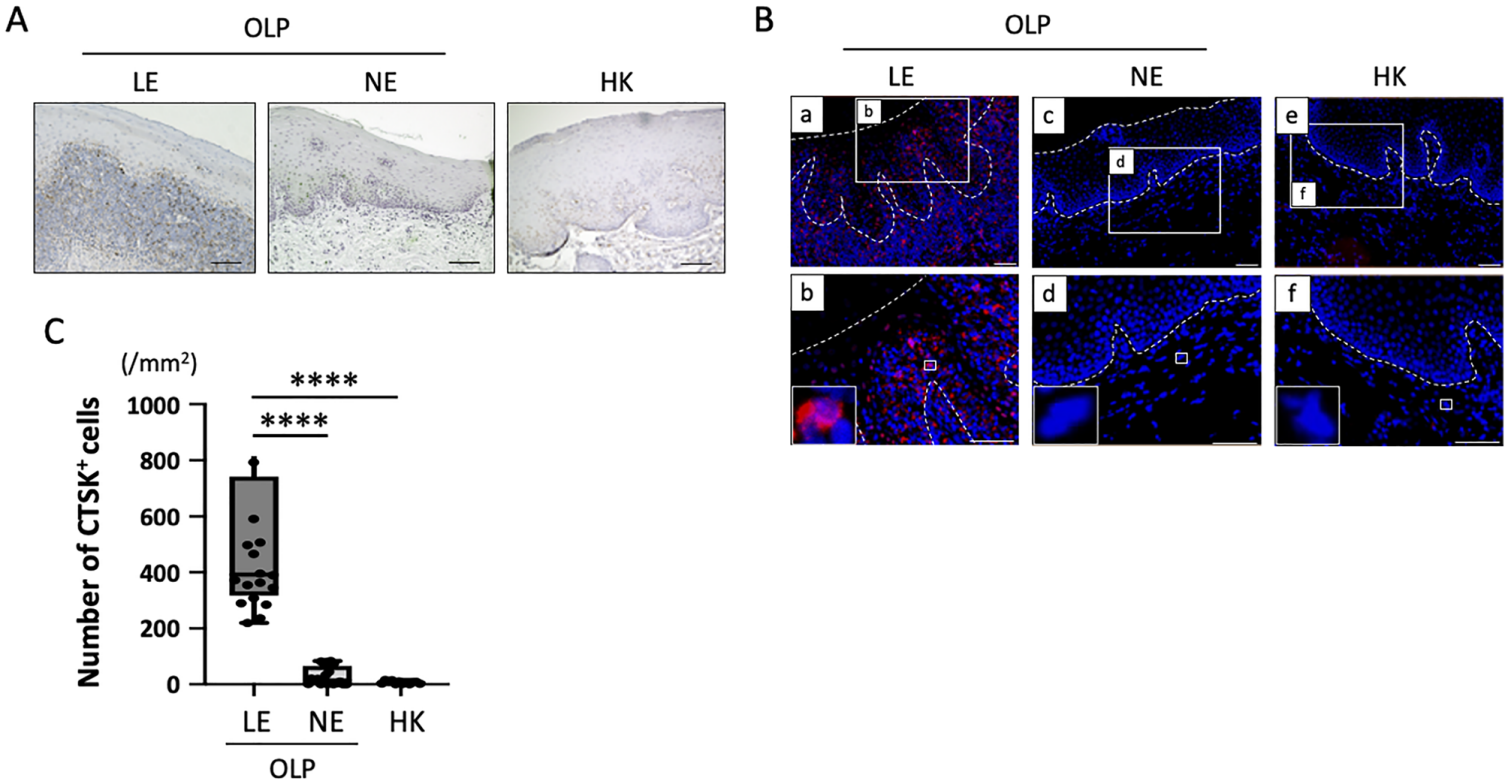
Expression and localization of cathepsin K (CTSK)-expressing cells in BM specimens from patients with OLP and hyperkeratosis (HK). (**A**) Representative images of CTSK-stained sections of BM specimens from patients with OLP and HK. Counterstaining was performed with Mayer’s hematoxylin (blue). Scale bars, 100 μm. (**B**) Representative images of** i**mmunofluorescence images of CTSK (red) in BM specimens from patients with OLP and HK. Outlined area indicates epithelium. Counterstaining was performed with DAPI (blue). Scale bars, 100 μm. (**C**) Number of CTSK-positive cells in BM specimens from patients with OLP (n = 20) and HK (n = 12) measured using TissueQuest software. Data are shown as box plots. Each box represents the upper and lower interquartile range. Lines inside the boxes represent the median. Symbols represent individual subjects. ***P < 0.001, ****P < 0.0001, by one-way ANOVA. See Fig. 1 for other definitions.

We next examined the expression and distribution of CTSK in BM specimens from patients with OLP and HK; representative immunohistochemical and immunofluorescence findings are shown in Fig. 2A and B, respectively. In patients with OLP, CTSK expression was strongly detected in the basal layer and just below in the lesion, but it was not detected in the normal site. In contrast, in patients with HK, CTSK expression was rarely seen in the lesions. Quantitative analysis in 20 patients with OLP and 12 with HK showed that the number of CTSK-positive cells in the LE from patients with OLP was significantly higher than that in the NE of OLP patients or the HK group (*P* < 0.0001) (Fig. 2C).

### Expression of TLR9 and identification of TLR9-expressing cells in OLP tissues

**Figure 3:**
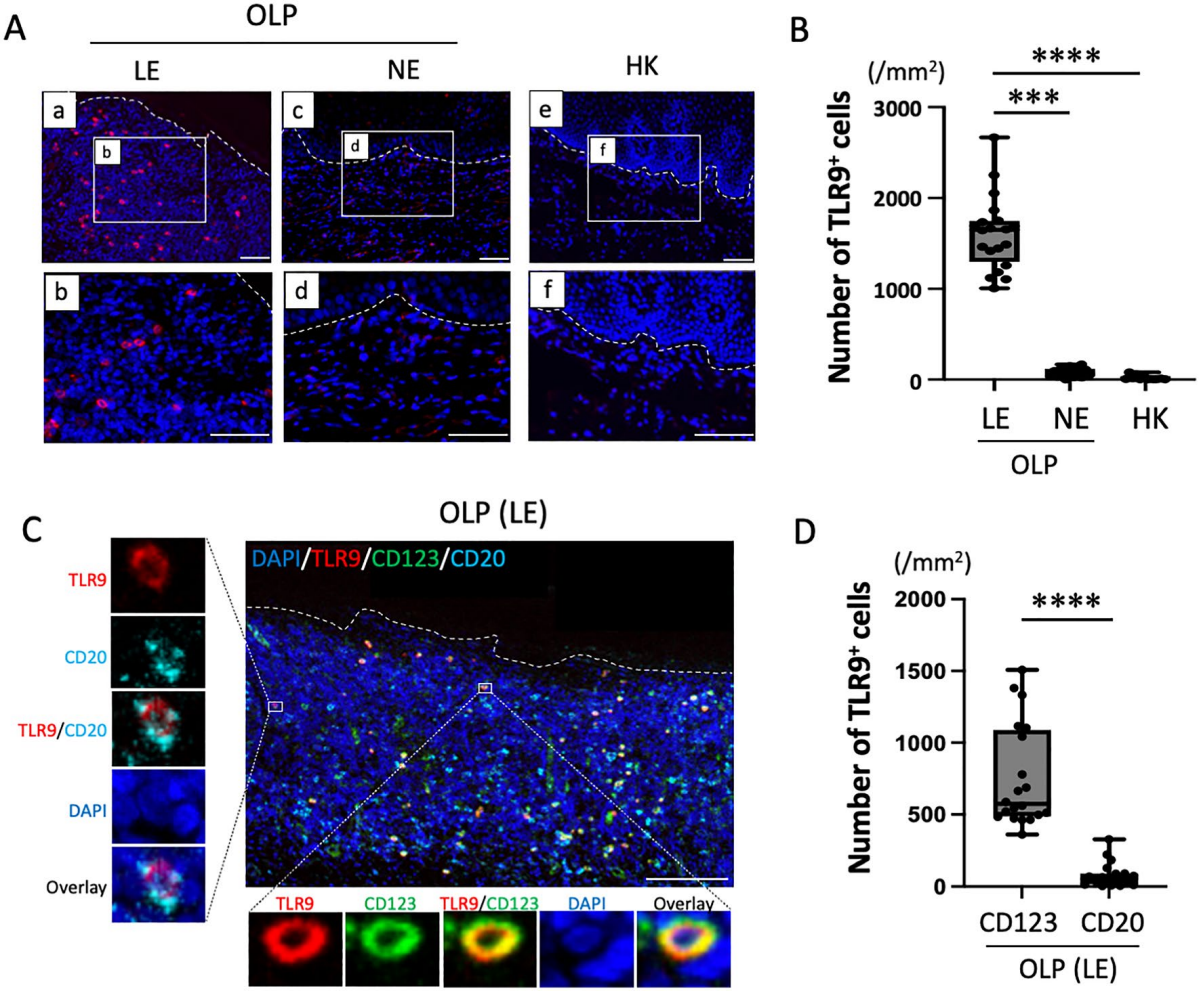
Identification and distribution of TLR9-expressing cells in BM specimens from patients with OLP. (**A**) Representative images of immunofluorescence staining of TLR9 (red) in BM specimens from patients with OLP and HK. Scale bars, 100 μm. (**B**) Number of TLR9-positive cells in BM specimens from patients with OLP (n = 20) and HK (n = 12). (**C**) Representative images of double immunofluorescence staining for TLR9 (red), CD20 (blue), and CD123 (green) in BM specimens from patients with OLP. Scale bars, 100 μm. (**D**) Number of TLR9-expressing CD20- and CD123-positive cells in BM specimens from patients with OLP (n = 20). In (**B**) and (**D**), data are shown as box plots. Each box represents the upper and lower interquartile range. Lines inside the boxes represent the median. Symbols represent individual subjects. ***P < 0.001, ****P < 0.0001, by one-way ANOVA (**B**) or Mann–Whitney *U* tests (**D**). See Fig. 1 for other definitions.

CTSK enhances TLR9 signaling and the production of Th17-related cytokines. We therefore examined the expression and distribution of TLR9 in BM specimens from patients with OLP and HK. TLR9 expression was strongly detected in subepithelial infiltrating cells under the LE from patients with OLP (Fig. 3A). Additionally, the number of TLR9-positive cells under the LE from patients with OLP was significantly higher than that under the NE of OLP patients (*P* < 0.001) or the HK group (*P* < 0.0001) (Fig. 3B).

TLR9 is expressed on B cells (CD20) and plasmacytoid DCs (pDCs) (CD123). We thus next examined TLR9-expressing cells in BM specimens using multi-immunofluorescence staining for TLR9, CD20, CD123, and DAPI. CD123-positive cells were mainly co-localized with TLR9 under the LE of OLP patients (Fig. 3C). Quantitative analysis in the LE from 20 patients with OLP revealed a significantly higher number of TLR9-expressing CD123-positive cells compared with the number of TLR9-expressing CD20-positive cells (*P* < 0.0001) (Fig. 3D).

### Th17-related cytokine production in pDCs via TLR9 signaling in vitro

**Figure 4:**
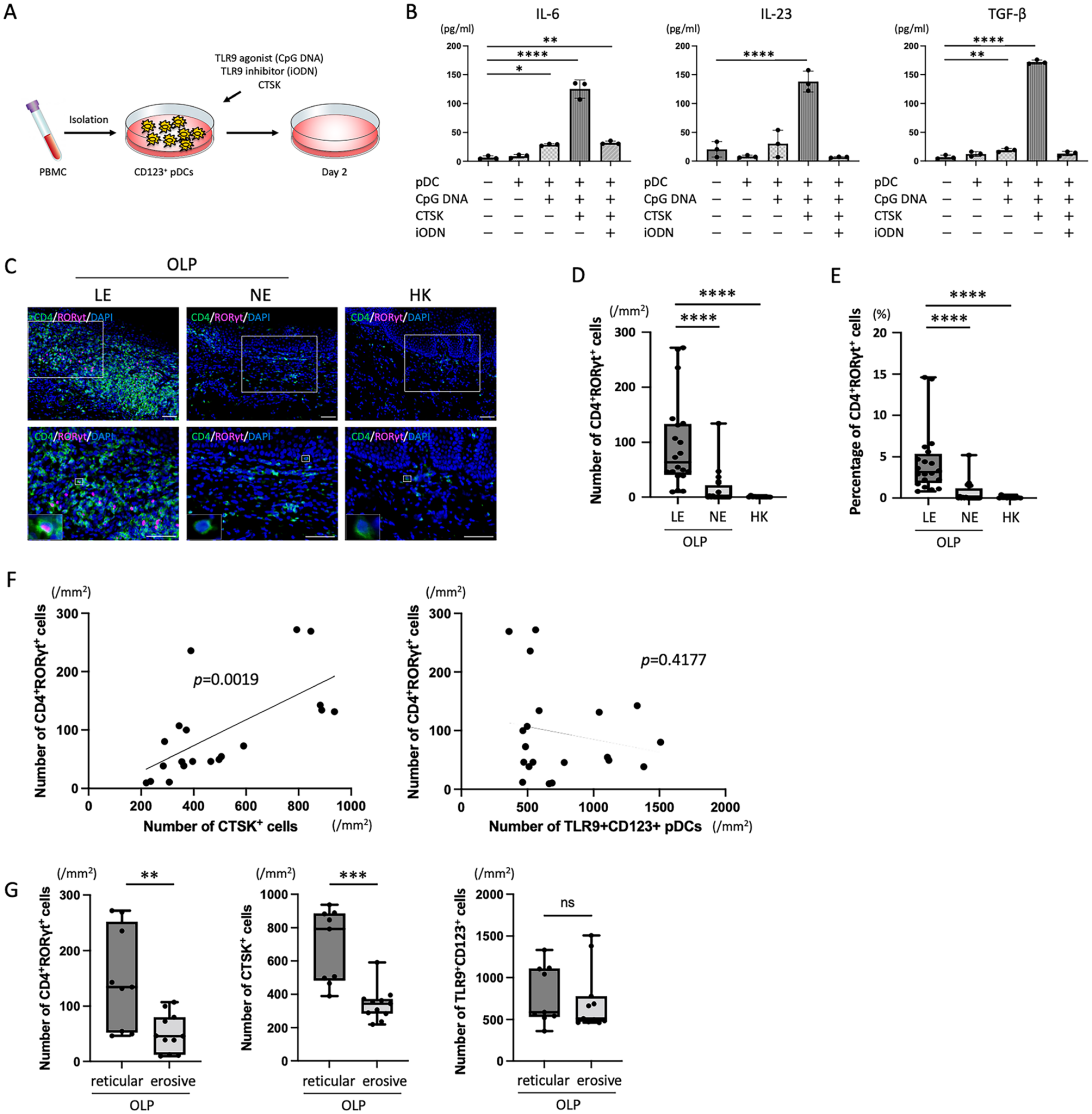
Association of CD123^+^ plasmacytoid dendritic cells (pDCs) with Th17 cell differentiation. (**A**) Schematic illustration of the experimental procedures; human CD123^+^ pDCs were extracted from peripheral blood mononuclear cells (PBMCs) and then stimulated by the Toll-like receptor 9 (TLR9) agonist CpG DNA, TLR9 inhibitor iODN, and/or CTSK in vitro. (**B**) Production of Th17-related cytokines (IL-6, IL-23, and TGF-β) in CD123^+^ pDCs stimulated with CpG DNA, iODN, and/or CTSK (n = 3/sample). Bars show the mean ± SD. Symbols represent individual subjects. *P < 0.05, **P < 0.01, by one-way ANOVA. (**C**) Representative images of immunofluorescence staining for CD4 (green), RORγt (pink) and DAPI (blue) in BM specimens from patients with OLP and HK. Scale bars, 100 μm. (**D**), (**E**) Quantification of CD4^+^RORγt^+^ Th17 cells in BM specimens from patients with OLP (n = 20) and HK (n = 12). (**F**) Correlation among the numbers of CTSK^+^ cells, TLR9^+^CD123^+^ pDCs, and CD4^+^RORγt^+^ Th17 cells in BM specimens from patients with OLP (n = 20). The correlation coefficients were determined by Spearman’s rank correlations. (**G**) Number of TLR9^+^CD123^+^ pDCs and CD4^+^RORγt^+^ Th17 cells in BM specimens from patients with reticular type OLP (n = 9) and erosive type OLP (n = 11). In** (D**), (**E**) and (**G**) data are shown as box plots. Each box represents the upper and lower interquartile range. Lines inside the boxes represent the median. Symbols represent individual subjects. **P < 0.01, ***P < 0.001, ****P < 0.0001 by one-way ANOVA (**D** and **E**) or Mann–Whitney *U* tests (**G**). See Fig. 1 for other definitions.

We next evaluated the in vitro effects of CTSK under stimulation of a TLR9 agonist (CpG DNA) or inhibitor (iODN) on Th17-related production derived from CD123^+^ pDCs (Fig. 4A). First, we confirmed the expression of TLR9 in CD123^+^ pDCs extracted from PBMCs as described in the Patients and Methods section. Next, Th17-related cytokines (IL6, IL23, and TGF-β) concentrations in the culture supernatant were measured with CTSK stimulation under stimulation of CpG DNA and/or iODN. IL-6 and TGF-β concentrations were significantly increased upon stimulation with CpG DNA and were further markedly increased by the addition of CTSK. IL-23 concentration was significantly increased only by the combination of CpG DNA and CTSK. Moreover, IL-6, IL-23, and TGF-β concentrations were significantly decreased in the presence of iODN (Fig. 4B). The TLR signaling pathway and the related DEGs are shown in Supplementary Fig. 3.

### Associations of Th17 cells with expression of CTSK and TLR9 in OLP tissues

To evaluate the subepithelial infiltration of Th17 cells in the lesion sites from patients with OLP and HK, multi-immunofluorescence staining for CD4, RORγt, and DAPI was performed (Fig. 4C). The number and percentage of CD4^+^RORγt^+^ Th17 cells were significantly higher in LE from OLP than in NE or HK groups (Fig. 4D,E and Supplementary Fig. 4). We then investigated the relationships among the numbers of CTSK^+^, TLR9^+^CD123^+^ pDCs, or CD4^+^RORγt^+^ Th17 cells in the lesion sites from patients with OLP. The number of CD4^+^RORγt^+^ Th17 cells showed a significant positive correlation with the number of CTSK^+^ cells in the 20 patients with OLP (Fig. 4F).

Next, we evaluated the associations among CTSK, pDCs, and Th17 cells with the clinical findings of OLP patients. The numbers of CTSK^+^ and CD4^+^RORγt^+^ Th17 cells in the reticular type was significantly higher than those in the erosive type (Fig. 4G).

### scRNA-seq analysis in BM specimens from patients with OLP.

**Figure 5:**
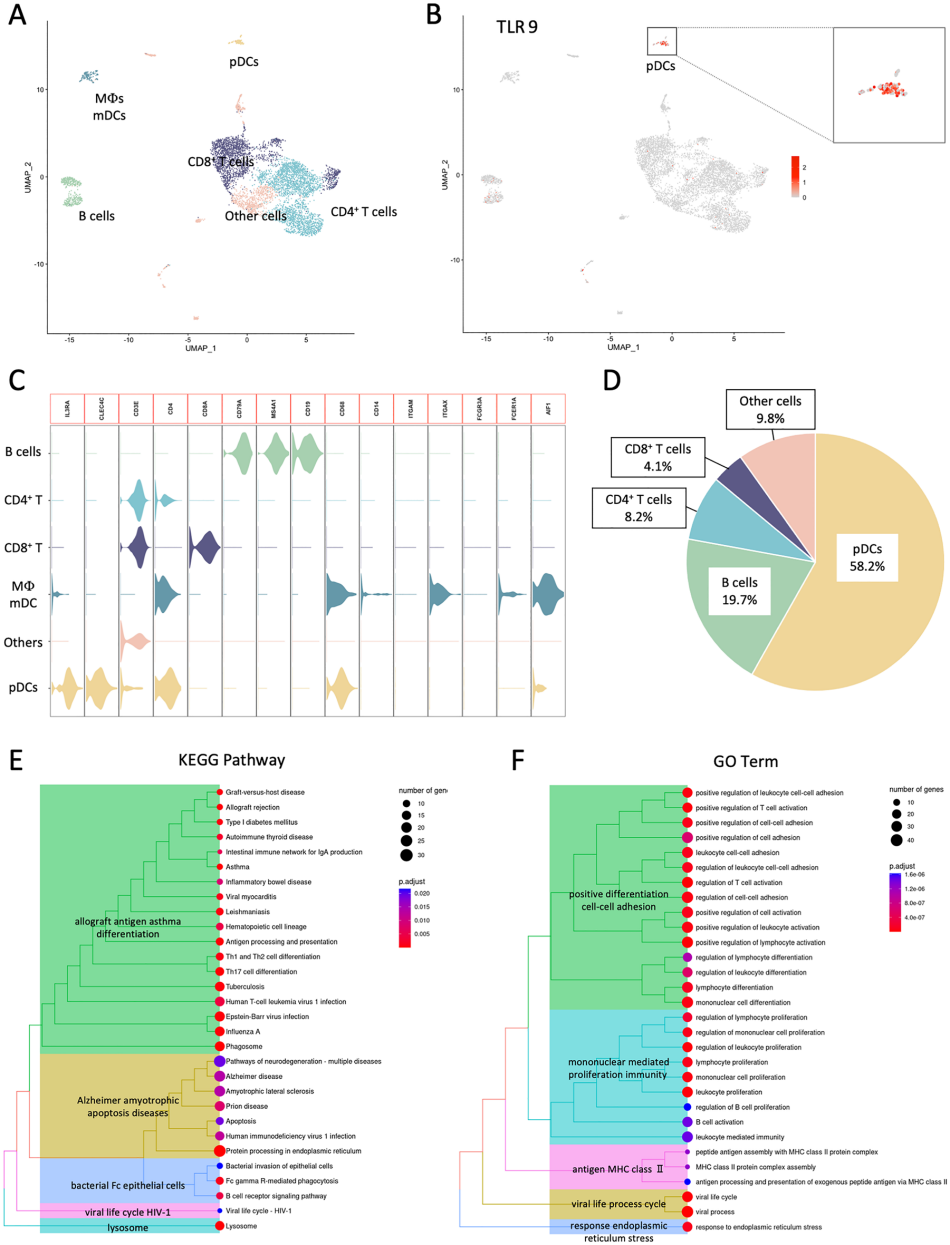
Single cell RNA-sequencing (scRNA-seq) analysis in BM specimens from patients with OLP. (**A**) Uniform manifold approximation and projection (UMAP) of CD45^+^ immune cells colored by cell types. (**B**) UMAP colored by TLR9 expression. (**C**) The violin plots showing the expression pattern of the selected cell markers in each of the clusters. (**D**) The percentage of CD45^+^ immune cells in TLR9^+^ cells. (**E**) KEGG pathways of upregulated DEGs in TLR9^+^ pDC compared with TLR9^−^ pDC. (**F**) GO analysis of DEGs upregulated in TLR9^+^ pDC compared with TLR9^−^ pDC.

We analyzed CD45^+^ immune cells extracted from BM specimens of patients with OLP by scRNA-seq (Fig. 5A). These cells were clustered depending on known marker (Fig. 5C). Since TLR9 strong expression was observed in the LE from OLP patients (Fig. 3A), we focused on TLR9 expression (Fig. 5B). Interestingly, pDCs accounted for the largest percentage of TLR9^+^ cells, consistent with the result of multi-immunofluorescence staining (Fig. 3C,D) and scRNA-seq (Fig. 5D). Moreover, we performed analysis of KEGG pathways and GO to identify DEGs that were upregulated in TLR9^+^ pDC compared with TLR9^−^ pDC, which revealed that the identified DEGs were involved in Th17 cell differentiation and positive regulation of T cell activation (Fig. 5E,F).

## Discussion

CD4^+^ Th cells, especially Th2 and Th17 cells, were shown to be involved in the immunopathogenesis of OLP through the induction of specific cytokines^6–8^. In our previous study, we focused on thymic stromal lymphopoietin (TSLP), a key cytokine that initiates and promotes the development of Th2 immune responses and that has been implicated in the progression of various allergic diseases. The receptor of TSLP (TSLPR) is mainly found in mDCs, which promote Th2 chemokine production via TSLP-TSLPR signaling^18^. We found that TSLP was distinctly expressed in/around the LE from OLP patients. Additionally, the number of TSLPR^+^CD11c^+^ mDCs showed a positive correlation with that of GATA3^+^ Th2 cells in OLP tissue. These results suggested that TSLPR^+^ mDCs contributed to the accumulation of Th2 cells and the development of OLP via TSLP production from the LE^6^.

Th17 cells are a subtype of CD4^+^ Th cells that express the retinoic acid receptor (RAR)-related orphan receptor (ROR)γt transcription factor and produce IL-17. In addition to IL-17, Th17 cells also produce IL-6, TGF-β, IL-21, IL-22, and IL-23^19^. Abnormal activity of Th17 cells has been implicated in the development of autoimmune disease including rheumatoid arthritis, multiple sclerosis, and Crohn’s disease^19,20^. Moreover, the IL-17A-triggered positive-feedback loop of IL-6 and TGF-β expression might promote Th17 cell expansion, which could be a general etiologic mechanism for various autoimmune diseases^21^. Several studies demonstrated the pathogenetic role of IL-17 in OLP on the basis of findings showing elevated serum IL-17 concentration and increased numbers of Th17 cells in the lesion and PBMCs from patients with OLP^8,22^. However, to the best of our knowledge, the mechanism for the differentiation to Th17 cells in LE in OLP has remained unclear.

In this study, we selectively extracted the LE of BM specimens by LCM, and CTSK was identified as one of epithelium-derived Th-immune molecules in the LE by DNA microarray. CTSK is a lysosome cysteine protease that is primarily expressed in osteoclasts and involved in bone remodeling and resorption^15,23^. In vivo studies demonstrated that CTSK plays an key role in the gene induction program regulated by TLR9 signaling, and CTSK-dependent TLR9 signaling in DCs contributes to cancer metastasis and autoimmune inflammation^16,24,25^. At least two subsets of DCs have been identified in humans: CD11c^+^CD123^−^ mDCs and CD11c^−^CD123^+^ pDCs. mDCs enhance Th1 and cytotoxic T lymphocyte responses via IL-6 and IL-12 production^26^. In contrast, pDCs participate in antiviral immune responses through Th17 immune reactions by the production of IFN-α^27^. Notably, pDCs expressing TLR9 abundantly produce Th17-related cytokines and prime Th17 cell development by stimulation of TLR9 agonist^28^. We found that the expressions of CTSK, TLR9, and RORγt in patients with OLP were significantly higher than those in the HK group, and there was a positive correlation between the numbers of CTSK^+^ and CD4^+^RORγt^+^ cells in OLP tissues. Furthermore, the numbers of these cells in early stage OLP (reticular type) were significantly higher than those in progressive stage OLP (erosive type), which suggests that epithelium-derived CTSK accelerates the production of Th17-related cytokines in CD123^+^ pDCs via TLR9 signaling. These results suggest that CTSK might be involved in the Th17-related inflammation of OLP, especially in early stage OLP.

**Figure 6:**
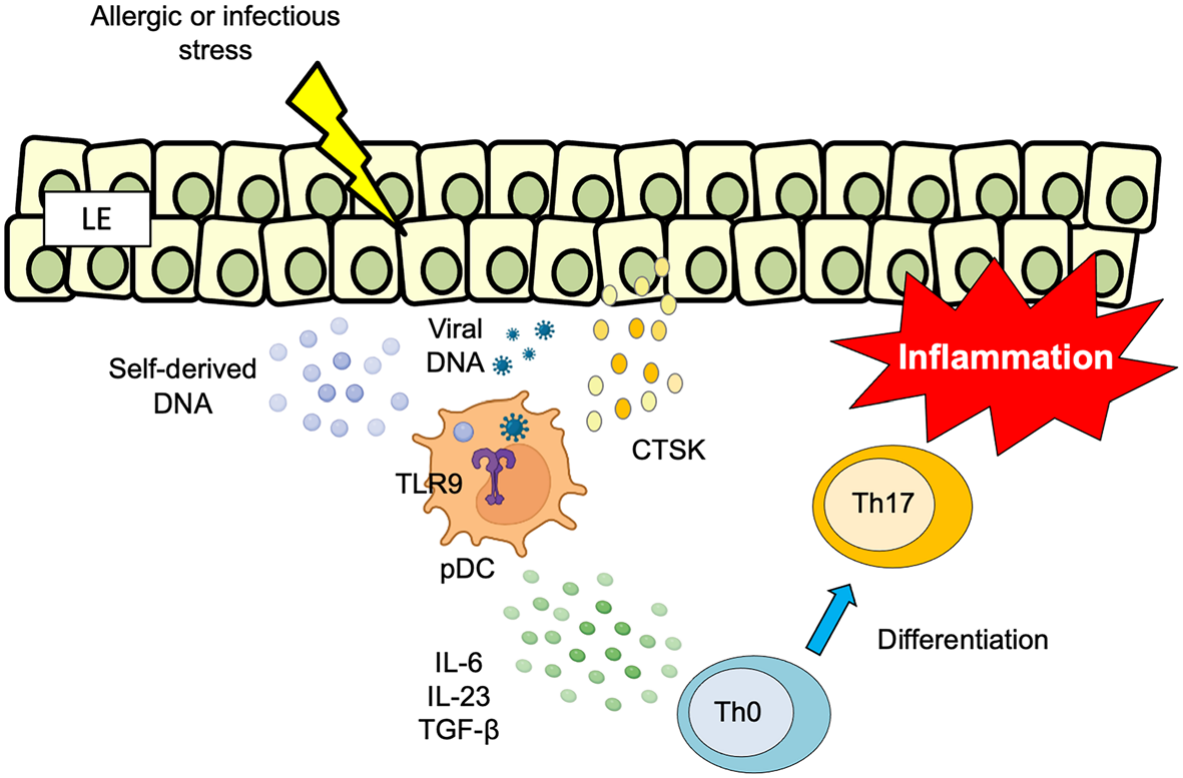
Schematic model of CTSK-induced TLR9 signaling in pDCs, leading to inflammation in OLP. TLR9 expressed on CD123-positive pDCs in subepithelial layers recognizes viral DNA. CTSK released from the LE activates TLR9 signaling in pDCs and their production of Th17-related cytokines, including IL-6, IL-23, and TGFβ, which leads to Th17-associated inflammation in OLP. See Fig. 1 for other definitions.

Our current data indicate that TLR9-expressing pDCs in the subepithelial layer recognize self-derived DNA or viral DNA. Activated pDCs produce several Th17-related cytokines (IL-6, IL-23, and TGF-β) via TLR9 signaling by CTSK released from the infected and injured LE, leading to chronic inflammation in OLP (Fig. 6). In a study using a mouse model of psoriasis, Hirai et al.^29^ found that CTSK promotes the production of Th17-related cytokines in mDCs in psoriasis via TLR7 signaling instead of TLR9 signaling. Our current study using single-cell RNA sequencing (scRNA-seq) revealed that TLR9^+^CD123^+^ pDCs were involved in Th17 cell differentiation. However, we did not confirm the direct effects of Th17 cell differentiation by pDCs via TLR9 signaling in OLP tissue.

The present study was conducted within a limited number of cases by single-center study. Further knowledge of immunological mechanism of OLP with an increasing number of cases by multi-center studies is necessary. In conclusion, OLP occasionally shows resistance to steroid therapy as the first-line therapy^30,31^ and in some occasions, can become cancerous. A more thorough understanding of the interaction between TLR9^+^CD123^+^ pDCs and CTSK in OLP may lead to the development of novel pharmacologic strategies to disrupt Th17 cell differentiation as a further means of suppressing the disease progression.

## Methods

### Study participants

The study design and methods were approved by the Institutional Review Board of the Center for Clinical and Translational Research of Kyushu University Hospital (IRB Serial Number: 2021-192 and 21108-00) and followed the tenets of the Declaration of Helsinki. The methods were carried out in accordance with the approved guidelines. All patients or their relatives gave their informed consent within the written treatment contract on admission and therefore prior to their inclusion in the study.

BM samples were obtained from patients who presented to the Department of Oral and Maxillofacial Surgery, Kyushu University Hospital between 2007 and 2021. A total of 20 patients with OLP (1 male and 19 female patients; mean age, 59.5 ± 13.4 years) and 12 patients with hyperkeratosis (5 male and 7 female patients; mean age, 65.3 ± 10.8 years) were included in this study. Diagnosis of OLP was made on the basis of the clinical phenotype and histopathological findings of HE–stained sections, including hyperkeratosis or parakeratosis, band-like lymphocytic (predominantly T-cell) infiltration underlying the epithelium. Moreover, all of OLP biopsy samples contain NE (Fig. 1A). On the other hand, patients with oral lichenoid lesions, oral cancer, autoimmune/rheumatic diseases, severe periodontal disease, and treated with steroids or any other immunosuppressants were clinically and pathologically excluded. The patients’ characteristics are listed in Supplementary Table 1.

### Tissue sampling by LCM

LCM was conducted using a Leica microdissection system (LMD6500; Leica Microsystems Japan, Tokyo, Japan). We selectively extracted the LE and NE from 20-μm-thick paraffin sections of BM specimens from three patients with OLP by LCM (Fig. 1B). We performed RNA extraction from microdissected samples and cDNA synthesis as previously described (6).

### Gene expression microarrays

We amplified and labeled complementary RNA using the Low Input Quick Amp Labeling Kit (Agilent Technologies, Santa Clara, CA, USA). The sample was then hybridized to SurePrint G3 Human Gene Expression Microarrays 8 60 K v2 (Agilent Technologies), following the manufacturer’s instructions. All hybridized microarray slides were scanned with an Agilent scanner. Relative hybridization intensities and background hybridization values were computed with Agilent Feature Extraction Software (ver. 9.5.1.1) as previously described^32,33^.

### Immunohistochemistry and quantitative analysis

The BM samples were cut into 4-μm-thick paraffin sections, and immunohistochemical staining with hematoxylin counterstaining was performed using anti-CD4 (Catalog No. ab133616; Abcam, Cambridge, MA, USA), anti-CD20 (Catalog No. ab78237; Abcam), anti-CD123 (Catalog No. NCL-L-CD123; Leica Biosystems, Newcastle, UK), anti-CTSK (Catalog No. sc-48353; Santa Cruz Biotechnology, Dallas, TX, USA), anti-RORγt (Catalog No. ACI 3208; Biocare Medical, Pacheco, CA, USA), and anti-TLR9 (Catalog No. ab134368; Abcam) as previously described^34^. For multi-color immunofluorescence staining, the 4-μm-thick paraffin sections were stained with the aforementioned antibodies using the Opal multiplex staining system (Opal 4-Color Manual IHC Kit, Catalog No. NEL810001KT).

For quantitative analysis, the stained tissue sections were scanned and automatically analyzed using TissueQuest software (TissueGnostics, Los Angeles, CA, USA). The principle of the quantitation method used in this study was previously described^35^. Cut-off values were defined in accordance with the positive controls, and numbers of single or double positive cells were calculated as cells per mm^2^.

### In vitro experiments

CD123^+^ pDCs were extracted from healthy peripheral blood mononuclear cells (PBMCs) using an EasySep Human Plasmacytoid DC Isolation Kit (Catalog No. 17977; StemCell Technologies, Vancouver, BC, Canada) (Supplementary Fig. 1). Cells were then stimulated with 3 μM of the TLR9 agonist CpG DNA (Catalog No. HC4037; Hycult Biotechnology, Uden, The Netherlands) and/or 400 nM of CTSK (Catalog No. SRP6561-5UG; Sigma-Aldrich, Saint Louis, MO, USA) and/or 10 μM of the TLR9 inhibitor iODN (Catalog No. IAX-200-050; Innaxon, Tewkesbury, UK) for 2 days. The supernatants were collected for further analysis.

### Flow cytometry

Collected cells were rinsed with eBioscience Flow Cytometry Staining Buffer (Thermo Fisher Scientific, Waltham, MA, USA) and then stained for 20 min at 37 °C in the dark with anti-human CD123-FITC (GHI/61; BioLegend). Cells were processed on a BD FACSVerse™ Flow Cytometer (Franklin Lakes, NJ, USA) and data were analyzed using BD FACSSuite Software (BD Biosciences).

### Enzyme-linked immunosorbent assay (ELISA)

The levels of IL-6, IL-23, and transforming growth factor (TGF)-β in cell culture supernatants were analyzed using a Human IL-6 ELISA Kit (Catalog No. KE00007; Proteintech, Rosemont, IL, USA), Human IL-23 ELISA Kit (Catalog No. 3457-1HP-2; Mabtech, Nacka Strand, Sweden), and Human TGF-β Quantikine ELISA Kit (Catalog No. DB100B; R&D Systems, Minneapolis, MN, USA) as previously described^36^.

### Dissociation of OLP tissue

The BM sample from OLP patients was dissociated into single-cell suspension via Multi Tissue Dissociation Kit 2 (Catalog No. 130-110-203; Miltenyi Biotec, Bergisch Gladbach, Germany) in gentleMACS Octo Dissociator with Heaters (Catalog No. 130-096-427; Miltenyi Biotec), then washed and resuspended in EasySep buffer (PBS + 2% FBS + 1 mM EDTA, Catalog No. 20144; STEMCELL). The cell aggregates or large particles in cell suspension were removed by MACS SmartStrainers (100 μm) (Catalog No. 130-098-463; Miltenyi Biotec) then the cell suspension was filtered into the 5 ml Polystyrene Round-Bottom Tube using its Cell-Strainer Cap (Catalog No. 352235; FALCON) and Red Blood Cell Lysis Solution (Catalog No. 130-094-183; Miltenyi Biotec) was used in the lysis of erythrocytes. After these steps, the collected cells were incubated with FcR Blocking Reagent (Catalog No. 130-059-901; Miltenyi Biotec) and PE anti-human CD45 Antibody (Catalog No. 304039; BioLegend) for 15 min at room temperature for downstream selection of CD45^+^ cells.

### CD45^+^ cells isolation and collection

EasySep Human PE Positive Selection Kit 2 (Catalog No. 17664; StemCell Technologies) and EasySep Magnet (Catalog No. 18000; StemCell Technologies) were used for the positive selection of CD45^+^ cells in the cell suspension. Then these CD45^+^ cells were centrifuged at 400 g for 5 min and stored at − 80 °C after resuspending in CELLBANKER 1 plus (Catalog No. 11912; ZENOGEN PHARMA, Fukushima, Japan) for scRNA-seq.

### scRNA-seq analysis

The quantity and survival rate of collection CD45^+^ cells were verified using a hemocytometer and adjusted to 10,000 single cells per sample then these cells were loaded in the 10 × Genomics Chromium device according to the protocol (Chromium Single Cell 5’ Reagent Kits, v2).

### Gene expression analysis

The output data of Illumina Hiseq was processed into align reads, generate feature-barcode matrix results using Cell Ranger 6.0. The downstream analysis was carried out in R (v4.2.0). The gene expression data of single-cell RNA sequencing was filtered basing on the criterion below: (1) Cells with mitochondrial content more than 20% and kinds of mRNA less than 200 would be seen as lysed cells and removed. (2) Cells with features (i.e., quantity of detected mRNA) more than 6000 would be seen as likely doublets and removed. Then the leukocyte phenotypes were assigned to different clusters, which were clustered via principal component analysis in the Seurat package (v4.0.6)^37^, using the marker genes: *CD14, CD68, ITGAX* and *AIF1* for macrophages and myeloid DCs (mDCs); *IL3RA* and *CLEC4C* for pDCs; *GNLY, NKG7* and *NCAM1* for NK cells; *CD19, MS4A1* and *CD79A* for B cells; *CD3E* and *CD4* for CD4^+^ T cells; *CD3E* and *CD8A* for CD8^+^ T cells. The assigned data was visualized by using uniform manifold approximation and projection (UMAP) dimensionality reduction^38^.

### Statistical analysis

Statistical analyses were performed using GraphPad Prism 9 (GraphPad Software, San Diego, CA, USA). The Mann–Whitney *U*-test, one-way ANOVA, and Spearman’s rank correlation were used for comparison of independent groups. A *P* value of < 0.05 was considered statistically significant.

### Ethics approval

The study design and methods were approved by the Institutional Review Board of Center for Clinical and Translational Research of Kyushu University Hospital (IRB Serial Number: 2021-192 and 21108-00).

### Consent to participate

All patients provided written informed consent prior to participation.

### Supplementary Information

Supplementary Figures.

Supplementary Table 1.
