# Supplementary material for: Toll-like receptor 9-positive plasmacytoid dendritic cells promote Th17 immune responses in oral lichen planus stimulated by epithelium-derived cathepsin K
Source: Sci Rep. 2023 Nov 7;13:19320. doi: 10.1038/s41598-023-46090-3 (PMC10630478; doi:10.1038/s41598-023-46090-3)
Supplement: Supplementary file 1 — Supplementary Figures. [file 41598_2023_46090_MOESM1_ESM.pdf]

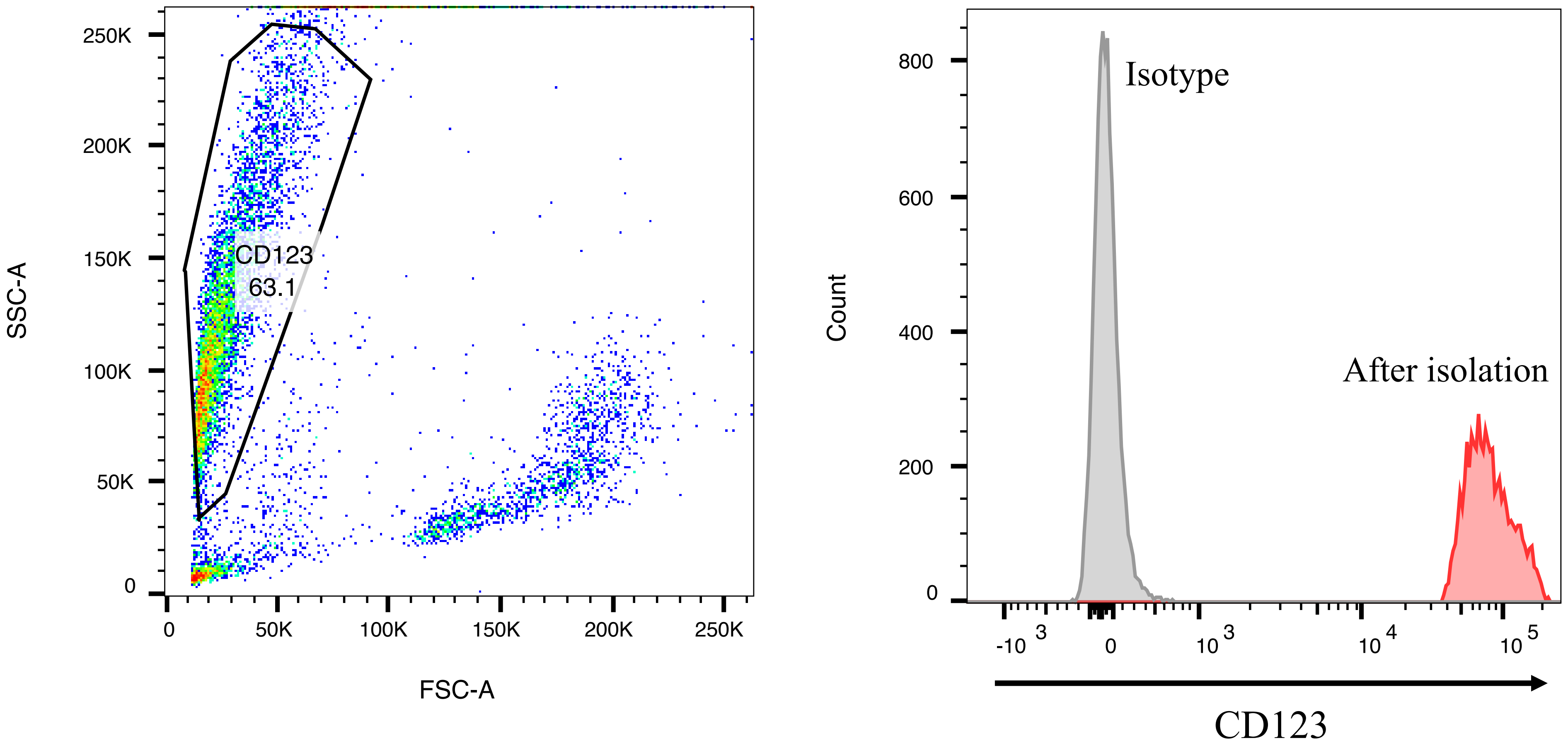


**Supplementary Figure 1.** Purity of the isolated CD123$^{+}$ cells were as determined by flow cytometric analysis. The detailed methods for isolating cells are described in the Materials and methods section.

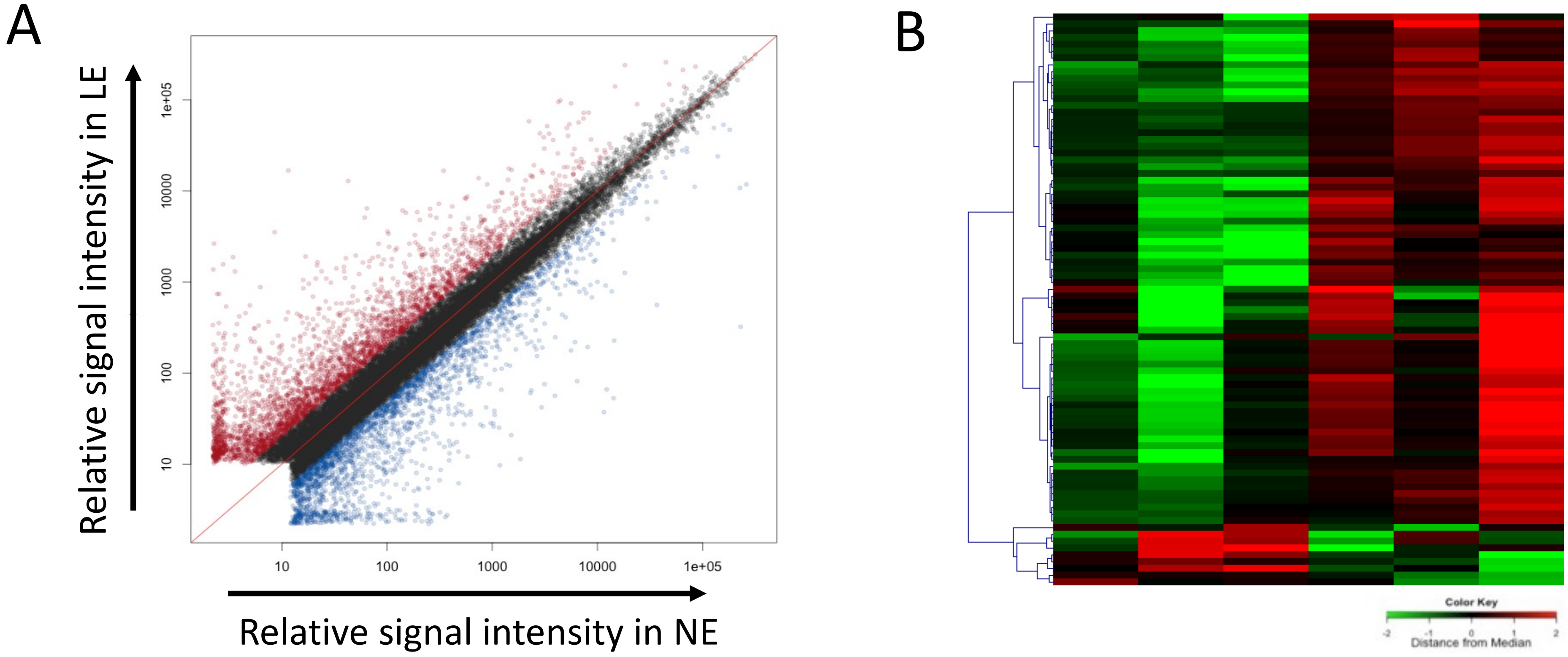


**Supplementary Figure 2.** Gene expression patterns in the lesional epithelium (LE) and normal epithelium (NE) from patients with oral lichen planus (OLP). **A,** Scatterplot analysis to elucidate and visualize the differences in gene expression between the LE and NE. Pink dots showed up-regulated genes and blue dots showed down-regulated genes in LE compared with NE. **B,** Heat map showed statistically significantly different in gene expression levels between the LE and NE. Colors represented the distance from the median values. Red blocks indicated high, green blocks low, and black blocks the median values.

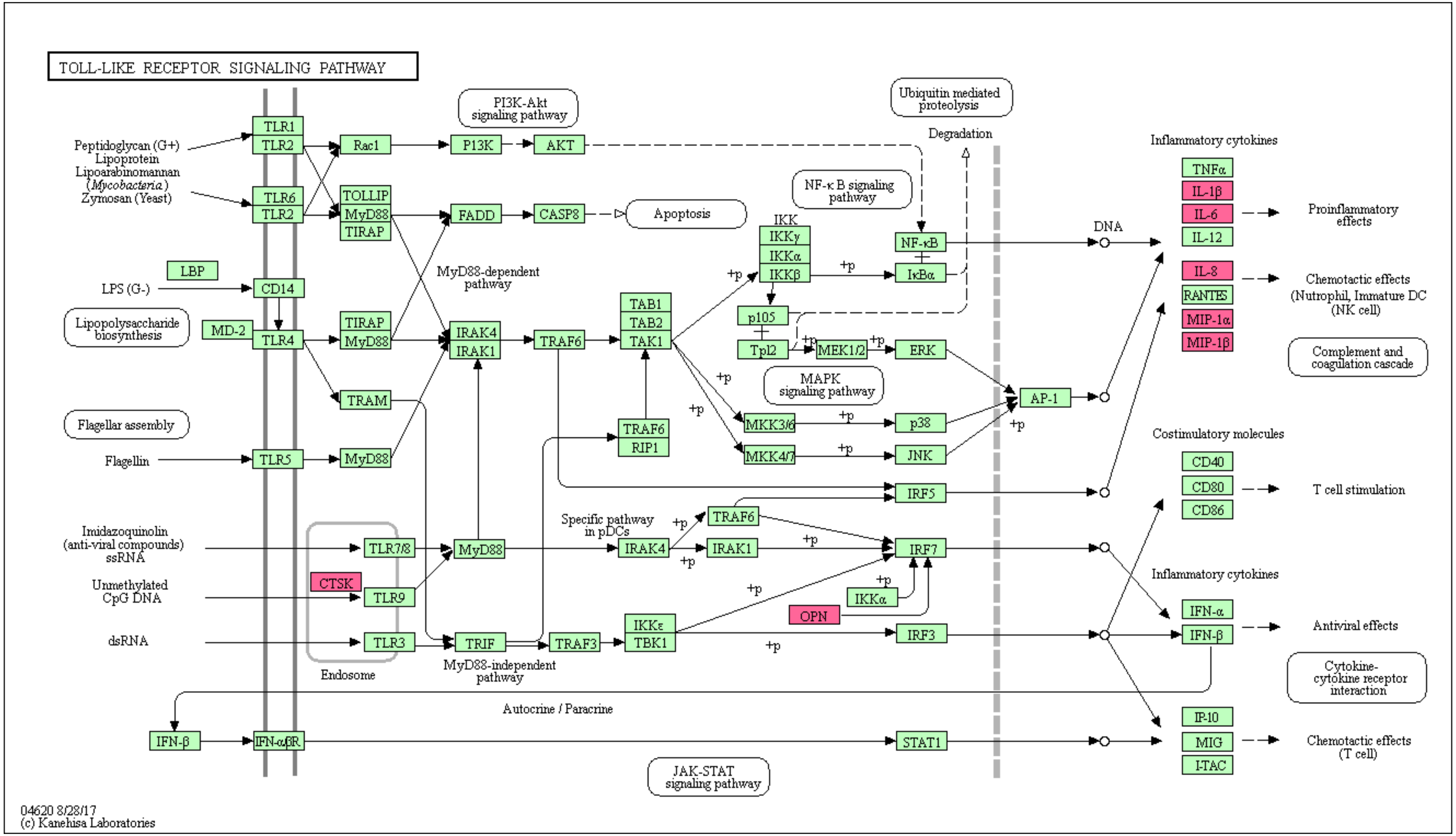


**Supplementary Figure 3.** Differentially expressed genes in Toll-like receptor signaling pathway from Kyoto Encyclopedia of Genes and Genomes (KEGG) pathways. Red columns represent DEGs located in up-regulated genes.

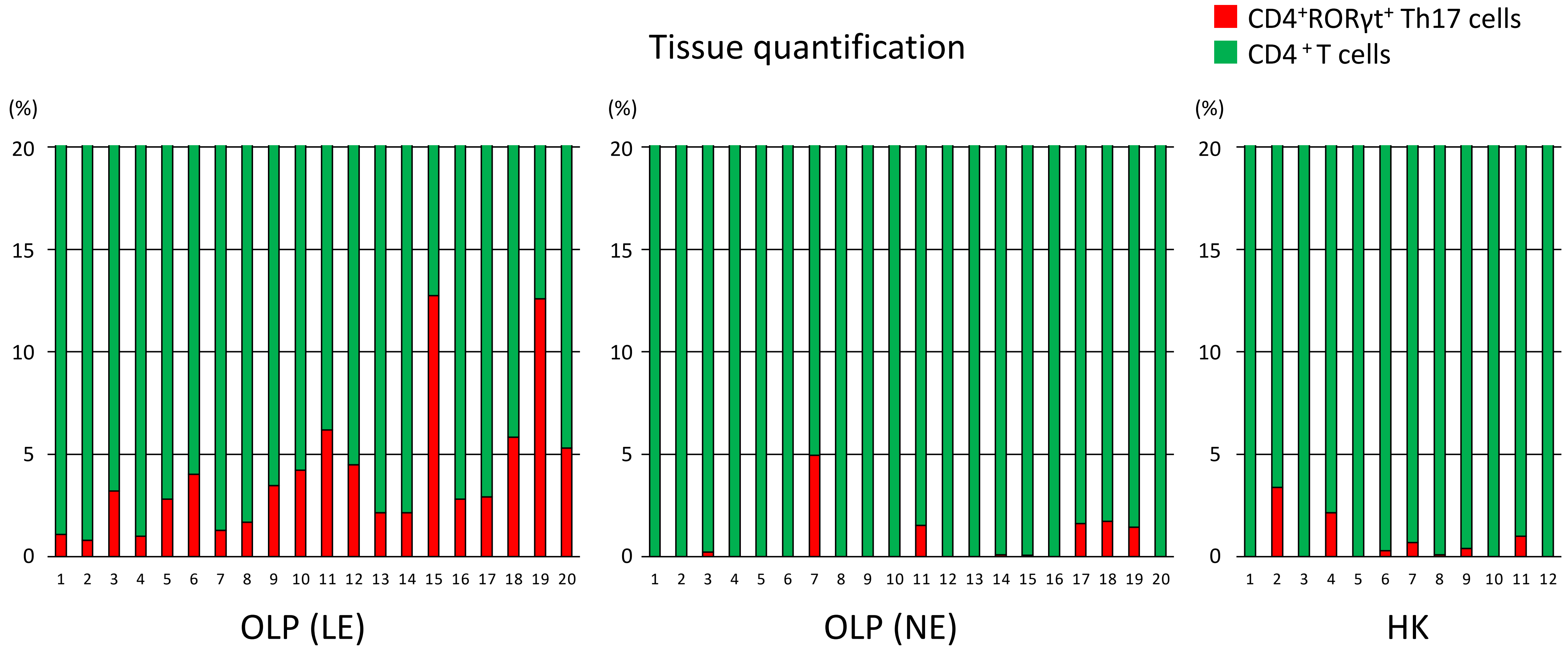


**Supplementary Figure 4.** Quantification of $CD4^{+}ROR\gamma t^{+}$ and $CD4^{+}ROR\gamma t^{-}$ cells in tissue biopsies from 20 patients with OLP (LE and NE) and 12 patients with hyperkeratosis (HK).
