# Supplementary material for: Toll-like receptor 9-positive plasmacytoid dendritic cells promote Th17 immune responses in oral lichen planus stimulated by epithelium-derived cathepsin K
Source: Sci Rep. 2023 Nov 7;13:19320. doi: 10.1038/s41598-023-46090-3 (PMC10630478; doi:10.1038/s41598-023-46090-3)
Supplement: Supplementary file 2 — Supplementary Table 1. [file 41598_2023_46090_MOESM2_ESM.doc]

**Supplementary Table 1.** Clinical findings of 20 patients with oral lichen planus included in this study.

| **No.** | **Age** | **Sex** | **Lesion localization** | **Lesion site**  **in BM** | **Clinical form** | **Metal**  **allergy** | **Complications** | **Medication** | **Smoking habit** | **Dental prosthesis** |
| --- | --- | --- | --- | --- | --- | --- | --- | --- | --- | --- |
| 1 | 62 | F | BM, Tongue | R/L | Reticular | − | None | − | − | + |
| 2 | 47 | F | BM, Gingiva | R/L | Reticular | + | None | − | − | + |
| 3 | 47 | F | BM, Lip | R/L | Erosive | − | HT | + | − | + |
| 4 | 77 | F | BM | R/L | Reticular | − | HT | + | − | + |
| 5 | 85 | F | BM, Lip | R/L | Reticular | − | HT, dyslipidemia | + | − | + |
| 6 | 52 | F | BM, Gingiva | R/L | Erosive | − | None | − | + | + |
| 7 | 54 | F | BM, Gingiva | R/L | Erosive | − | DM, dyslipidemia | + | − | + |
| 8 | 62 | F | BM, Gingiva | R | Erosive | − | HT | + | − | + |
| 9 | 44 | F | BM, Gingiva | R/L | Reticular | − | None | − | − | + |
| 10 | 79 | F | BM, Tongue, Lip | R/L | Erosive | − | Osteoporosis | + | − | + |
| 11 | 79 | F | BM, Gingiva | R/L | Reticular | − | HT, osteoporosis | − | − | + |
| 12 | 29 | F | BM, Gingiva | R/L | Erosive | + | None | − | − | + |
| 13 | 56 | F | BM, Lip | R/L | Erosive | − | HT, DM | + | + | + |
| 14 | 47 | F | BM | R/L | Erosive | − | None | − | − | + |
| 15 | 65 | F | BM | R | Reticular | − | None | − | − | + |
| 16 | 60 | F | BM, Gingiva | R/L | Reticular | − | None | − | + | + |
| 17 | 55 | F | BM, Lip | R/L | Erosive | − | None | − | − | + |
| 18 | 63 | F | BM, Gingiva | R/L | Erosive | − | HT, osteoporosis | + | − | + |
| 19 | 59 | M | BM | R/L | Erosive | − | HT | + | − | + |
| 20 | 67 | F | BM | L | Reticular | − | Dyslipidemia | + | − | + |

Abbreviations: BM, buccal mucosa; HT, hypertension; DM, diabetes mellitus
